# Evaluating a variety of text-mined features for automatic protein function prediction with GOstruct

**DOI:** 10.1186/s13326-015-0006-4

**Published:** 2015-03-18

**Authors:** Christopher S Funk, Indika Kahanda, Asa Ben-Hur, Karin M Verspoor

**Affiliations:** Computational Bioscience Program, University of Colorado School of Medicine, Aurora, 80045 CO USA; Department of Computer Science, Colorado State University, Fort Collins, 80523 CO USA; Department of Computing and Information Systems, University of Melbourne, Parkville, 3010 Victoria Australia; Health and Biomedical Informatics Centre, University of Melbourne, Parkville, 3010 Victoria Australia

**Keywords:** Text mining, Protein function prediction, Biomedical concept recognition

## Abstract

Most computational methods that predict protein function do not take advantage of the large amount of information contained in the biomedical literature. In this work we evaluate both ontology term co-mention and bag-of-words features mined from the biomedical literature and analyze their impact in the context of a structured output support vector machine model, GOstruct. We find that even simple literature based features are useful for predicting human protein function (F-max: Molecular Function =0.408, Biological Process =0.461, Cellular Component =0.608). One advantage of using literature features is their ability to offer easy verification of automated predictions. We find through manual inspection of misclassifications that some false positive predictions could be biologically valid predictions based upon support extracted from the literature. Additionally, we present a “medium-throughput” pipeline that was used to annotate a large subset of co-mentions; we suggest that this strategy could help to speed up the rate at which proteins are curated.

**Electronic supplementary material:**

The online version of this article (doi:10.1186/s13326-015-0006-4) contains supplementary material, which is available to authorized users.

## Introduction

Characterizing the functions of proteins is an important task in bioinformatics today. In recent years, many computational methods to predict protein function have been developed to help understand functions without performing costly experiments. Most computational methods use features derived from sequence, structure or protein interaction databases [1]; very few take advantage of the wealth of unstructured information contained in the biomedical literature. Because little work has been conducted using the literature for function prediction, it is not clear what type of text-derived information will be useful for this task or the best way to incorporate it. In this work, we evaluate two different types of literature features, co-occurrences of specific concepts of interest as well as a bag-of-words model, and assess the most effective way to combine them for automated function prediction. We also provide many examples of the usefulness of literature features for verification or validation of automated predictions.

## Background

Literature mining has been shown to have substantial promise in the context of automated function prediction, although there has been limited exploration to date [2]. The literature is a potentially important resource for this task, as it is well known that the published literature is the most current repository of biological knowledge and curation of information into structured resources has not kept up with the explosion in publication [3]. A few teams from the first Critical Assessment of Functional Annotation (CAFA) experiments [1] used text-based features to support prediction of Gene Ontology (GO) functional annotations [4].

Wong and Shatkay [5] was the only team in CAFA that used exclusively literature-derived features for function prediction. They utilized a *k*-nearest neighbor classifier with each protein related to a set of predetermined characteristic terms. In order to have enough training data for each functional class, they condensed information from all terms to those GO terms in the second level of the hierarchy, which results in only predicting 34 terms out of the thousands in the Molecular Function and Biological Process sub-ontologies. Recently, there has been more in-depth analysis into how to use text-based features to represent proteins from the literature without relying on manually annotated data or information extraction algorithms [6]. This work explored using abstracts along with unigram/bigram feature representation of proteins.

Another team, Björne and Salakoski [7], utilized events, specifically molecular interactions, extracted from biomedical literature along with other types of biological information from databases; they focused on predicting the 385 most common GO terms.

The work we presented in the first CAFA [8] is on a different scale from these previous efforts, and integrates information relevant for predicting protein function from a range of sources. We utilize as much of the biomedical literature as possible and are able to make predictions for the entire Gene Ontology, thanks to a structured output support vector machine (SVM) approach called GOstruct [9]. We found in that previous work that features extracted from the literature alone approach performance of many commonly used features from non-literature sources, such as protein-protein interactions derived from a curated resource. However, we used only concept co-occurrence features – focusing on simple, scalable features – leaving open many questions about the best strategy for representing the literature for the task of automated protein function prediction.

In this work, we therefore explore a variety of text-mined features, and different ways of combining these features, in order to understand better the most effective way to use literature features for protein function prediction. We have extended our workshop paper [10] by refining the enhanced GO extraction rules, performing more extensive analysis of the data at the functional class level, and extending validation through manual curation using a “medium-throughput” curation pipeline. We again explore these questions in the context of the structured output SVM model, GOstruct.

## Methods

**Figure 1:**
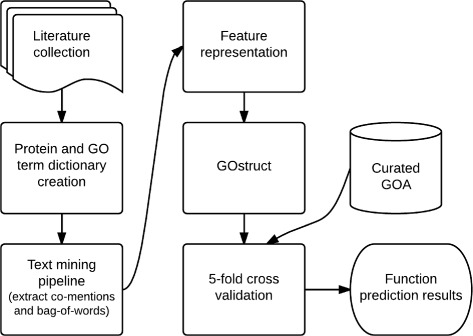
**Overview of the experimental setup used for function prediction.**

An overview of our experimental setup can be seen in Figure 1 with more specific details about each process following.

### Data

We extracted text features from two different literature sources: (1) 13,530,032 abstracts available from Medline on October 23, 2013 with both a title and abstract text and (2) 595,010 full-text articles from the PubMed Open Access Collection (PMCOA) downloaded on November 6, 2013. These literature collections were processed identically and features obtained from both were combined. Gold standard Gene Ontology annotations for both human and yeast genes were obtained from the Gene Ontology Annotation (GOA) data sets [11]. Only annotations derived experimentally were considered (evidence codes EXP, IDA, IPI, IMP, IGI, IEP, TAS). Furthermore, the term Protein Binding (GO:0005515) was removed due to its broadness and overabundance of annotations. The human gold standard set consists of over 13,400 proteins annotated with over 11,000 functional classes while the yeast gold standard set consists of over 4,500 proteins annotated with over 6,500 functional classes. Even though the gold standard sets are large, only proteins where there is enough training data will produce predictions. Additionally, to produce meaningful area under the curve (AUC) scores only GO terms with at least 10 annotations in the gold standard are considered as possible prediction targets; this corresponds to 509 Molecular Function classes, 2,088 Biological Process classes, and 345 Cellular Component classes.

### Literature features

Two different types of literature features were extracted and evaluated, co-mentions and bag of words. Co-mentions are mentions of both a specific protein and concept from the Gene Ontology that co-occur with a specified span of text; they represent a simple knowledge-directed approach to represent the information contained within the biomedical literature. Another representation of biomedical information is to relate proteins to words mentioned in the surrounding context; this is a knowledge-free approach because we are not grounding what we relate to proteins into some ontology, but only strings.

#### Text-mining pipeline

A pipeline was created to automatically extract the two different types of literature features using Apache UIMA version 2.4 [12]. Whole abstracts were provided as input and full-text documents were provided one paragraph at a time. The pipeline consists of splitting the input documents into sentences, tokenization, and protein entity detection through LingPipe trained on CRAFT [13], followed by mapping of protein mentions to UniProt identifiers through a protein dictionary. Then, Gene Ontology (GO) terms are recognized through dictionaries provided to ConceptMapper [14]. Finally, counts of GO terms associated with proteins, and sentences containing proteins, are output. A modified pipeline to extract proteins, GO terms, or any entity from an ontology file from text is available at http://bionlp.sourceforge.net/nlp-pipelines/. Details of the individual steps are provided below.

#### Protein mention extraction

The protein dictionary consists of over 100,000 protein targets from 27 different species, all protein targets from the CAFA2 competition (http://biofunctionprediction.org). To increase the ability to identify proteins in text, synonyms for proteins were added from UniProt (UniProt Consortium 2008) and BioThesaurus version 0.7 [15].

#### Gene ontology term extraction

The best performing dictionary-based system and parameter combination for GO term recognition identified in previous work was used [16]. ConceptMapper (CM) is highly configurable dictionary lookup system that is a native UIMA component. CM is highly configurable through the use of many parameters. The list of parameters used to extract GO terms is in Additional file 1.

Two different dictionaries were provided to CM to extract Gene Ontology mentions from text: original and enhanced. Both dictionaries are based on GO from 2013-11-13. The original directly utilizes GO terms and synonyms, with the exception that the word “activity” was removed from the end of ontology terms. The enhanced dictionary augments the original dictionary with additional synonyms for many GO concepts. Rules were manually created by examining variation between ontology terms and the annotated examples in a natural language corpus. This enhanced dictionary improved GO recognition F-measure performance on CRAFT corpus [13,17] by 0.1 (from 0.49 to 0.59), through application of term transformation rules to generate synonyms.

A simple rule deals with the many GO terms of the form “*X* metabolic process”, which we have observed often do not occur literally in published texts. For example, for term GO:0043705, “cyanophycin metabolic process” synonyms of “cyanophycin metabolism” and “metabolism of cyanophycin” are generated. It is also noted that most of the terms in GO are nominals, so it is important to generate part of speech variants. There are also many “positive regulation of *X*” terms; not only will we generate synonyms of “positive regulation of” such as “stimulation” and “pro”, but if there exist inflectional and derivational variants of *X* we can also substitute that in. For example, “apoptotic stimulation” and “pro-apoptotic” are added for “positive regulation of apoptosis” (GO:0043065). The version of the enhanced dictionary differs from the dictionary originally used for CAFA2, as described in [10].

#### Co-mentions

Co-mentions are based on co-occurrences of entity and ontology concepts identified in the literature text. This approach represents a targeted knowledge-based approach to feature extraction. The co-mentions we use here consist of a protein and Gene Ontology term that co-occur anywhere together in a specified span. While this approach does not capture relations as specific as an event extraction strategy [7], it is more targeted to the protein function prediction context as it directly looks for the GO concepts of the target prediction space. It also has higher recall since it doesn’t require an explicit connection to be detected between the protein and the function term.

For these experiments, we considered two spans: sentence and non-sentence. Sentence co-mentions are two entities of interest seen within a single sentence while non-sentence co-mentions are those that are mentioned within the same paragraph/abstract, but not within the same sentence. The number of co-mentions extracted for human and yeast proteins using both dictionaries can be seen in Table 1. For human proteins, the enhanced dictionary identifies 1,500 more GO terms than the original dictionary, which, leads to a 35% increase in the number of co-mentions identified (∼56 million more).

**Table 1:** **Statistics of co-mentions extracted from both Medline and PMCOA using the different dictionaries for identifying GO terms**

**Human**					
**Dictionary**	**Span**	**Unique proteins**	**Unique GO terms**	**Unique co-mentions**	**Total co-mentions**
Original	sentence	12,826	14,102	1,473,579	25,765,168
	non-sentence	13,459	17,231	3,070,466	147,524,964
	combined	13,492	17,424	3,222,619	173,289,862
Enhanced	sentence	12,998	15,415	1,839,360	33,199,284
	non-sentence	13,513	18,713	3,725,450	196,761,554
	combined	13,536	18,920	3,897,951	229,960,838
**Yeast**					
**Dictionary**	**Span**	**Unique proteins**	**Unique GO terms**	**Unique co-mentions**	**Total co-mentions**
Original	sentence	5,016	9,471	317,715	2,945,833
	non-sentence	5,148	12,582	715,363	18,142,448
	combined	5,160	12,819	748,427	21,088,281
Enhanced	sentence	5,063	12,877	414,322	3,853,994
	non-sentence	5,160	13,769	901,123	23,986,761
	combined	5,167	14,018	939,743	27,840,755

#### Bag-of-words

Bag-of-words (BoW) features are commonly used in many text classification tasks. They represent a knowledge-free approach to feature extraction. For these experiments, proteins are associated to words from sentences in which they were mentioned. All words were lowercased and stop words were removed, but no type of stemming or lemmatization was applied.

#### Feature representation

The extracted literature information is provided to the machine learning framework as sets of features. Each protein is represented as a list of terms, either Gene Ontology or words, along with the number of times the term co-occurs with that protein in all of the biomedical literature. An example entry from the co-mention features is as follows: “Q9ZPY7, co_GO:0003675=6, co_GO:0005623=2, co_GO:0009986=2, co_GO:0016020=2…”. We utilize a sparse feature representation and only explicitly state the non-zero features for both co-mentions and BoW.

### Experimental setup

We evaluate the performance of literature features using the structured output SVM approach GOstruct [9]. GOstruct models the problem of predicting GO terms as a hierarchical multi-label classification task using a single classifier. As input, we provide GOstruct with different sets of literature features for each protein, as described above, along with the gold standard GO term associations of that protein, used for training. From these feature sets, GOstruct learns patterns associating the literature features to the known functional labels for all proteins in the training set. Given a set of co-occurring terms for a single protein, a full set of relevant Gene Ontology terms can be predicted. In these experiments, we use no additional resource beyond the literature to represent proteins.

GOstruct provides confidence scores for each prediction; therefore, all results presented in this paper are based upon the highest F-measure over all sets of confidence scores, F-max [1]. Precision, recall, and F-max are reported based on evaluation using 5-fold cross validation. To take into account the structure of the Gene Ontology, all gold standard annotations and predictions are expanded via the ‘true path rule’ to the root node of GO. The ‘true path rule’ states that ‘the pathway from a child term all the way up to its top-level parent(s) must always be true’. We then compare the expanded set of terms. (This choice of comparison impacts the interpretations of our results, which is discussed further below). All experiments were conducted on both yeast and human.

Note that the ‘true path rule’ is only utilized during the evaluation of features through machine learning system (as discussed in *Impact of evaluation metric on performance*). All numbers reported about the performance and predictions made by the machine learning system have the rule applied, while numbers strictly referring to counts of co-mentions mined from the literature do not.

### Human evaluation of co-mentions

To support evaluation of the accuracy of the co-mention features, we sampled a number of them and asked a human assessor to rate each one as “good” (True Positive) or “bad” (False Positive), i.e., whether or not it captures a valid relationship. To assess accuracy of co-mentions as a whole, 1,500 sentence co-mentions were randomly sampled from the 33.2 million co-mentions for annotation. Additionally, three smaller subsets of co-mentions of specific functional classes, totaling about 500 co-mentions, were selected for annotation to assess accuracy of sentence co-mentions for specific functional classes. In total, there were around 3,000 full sentences annotated.

To enable fast annotation of this rating, we developed an approach that allows for “medium-throughput” manual annotation of co-mentions, about 60-100 per hour. The sentence co-mentions are transformed to brat rapid annotation tool (http://brat.nlplab.org/) format. The annotator views both the identified protein and functional concept in differing colors within the context of the entire sentence. The annotator is only required to connect them with a single relationship, either “Good-Comention” or “Bad-Comention”. The annotator was instructed to view the labeled protein and GO concept as correct and to only annotate “Good-Comention” when there exists a relationship between the specified entities. While a relationship may exist between the annotated GO category and another exact mention of the labeled protein, that would be considered incorrect for the purposes of this annotation, i.e., it is a decision relative to individual mentions of the protein in a specific textual context. We utilized these annotations to assess quality of a random set of co-mentions and also to label subsets of co-mentions containing particular functional concepts.

## Results and discussion

### Exploring the use of co-mention features

We mined co-mentions from two different text spans and explore four different ways to use them.

1. only using sentence co-mentions
2. only using non-sentence co-mentions
3. combining counts from sentence and non-sentence co-mentions into one feature set in the input representation
4. using two separate feature sets for sentence and non-sentence co-mentions

The spans were explained in more detail above, under the *Co-mentions* section.

**Figure 2:**
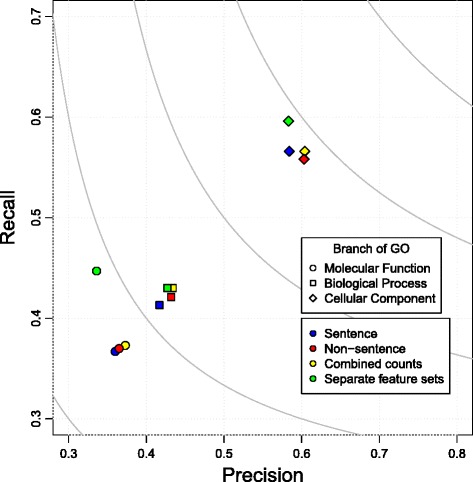
**Precision, recall, and F-max performance of four different co-mention feature sets on function prediction.** Better performance is to the upper-right and the grey iso bars represent balance between precision and recall. Diamonds – Cellular Component, Circle – Biological Process, Square – Molecular Function.

The performance of these four different strategies for combining the co-mention features for the enhanced dictionary can be seen in Figure 2. Each branch of GO is predicted and evaluated separately, but the way to combine features is the same for all branches. Similar trends are seen with the original dictionary (data not shown).

Using the two types of co-mentions as two separate feature sets provide the best performance on all branches of GO (see green shapes in Figure 2). These two types of co-mentions encode different but complementary information and the classifier is able to build a better model by considering them separately.

We utilized our “medium-throughput” human annotation pipeline and curated 1,500 randomly sampled sentence co-mentions; we found that ∼30% (441 out of 1,500) appeared to correctly relate the labeled protein with the labeled function. From these results it seems that sentence co-mentions contain a high false positive rate, most likely due to many mentions of proteins or GO concepts within a single sentence. Methods for filtering sentences that contain ambiguous mentions, due to both ambiguous protein names and many annotations within sentences containing complex syntactic structure, are still to be explored. Additionally, more complicated relationship or event detection would reduce the number of false positives seen and provide the classifier with higher quality sentence co-mentions, but significantly reduce the total number of identified co-mentions. It is unclear which method would be preferred for function prediction features.

Interestingly, non-sentence co-mentions perform better than sentence co-mentions. This goes against intuition, as co-mentions within a sentence boundary act as a proxy to a relationship between the protein and its function. However, it was seen in Bada et al. [18] that often function annotations do not occur within a sentence boundary with the corresponding protein. While coreference resolution may be required to correctly resolve such relationships, capturing function concepts in close proximity to a protein appears to be a useful approximation. This could be the reason why non-sentence co-mentions perform better. Based upon these results, from now on, when we say “co-mention features” we are referring to using both sentence and non-sentence as separate feature sets but within the same classifier.

To establish a baseline we utilized the co-mentions themselves as a classifier; the co-mentions are used as the final predictions of the system. We performed evaluations using both original and enhanced co-mentions. Results from combining counts between sentence and non-sentence co-mentions are presented in Table 2. The baseline leads to very low precision for all branches but we do see impressive levels of recall. This signifies that information from the literature is able to capture relevant biological information, but because we are able to identify many different co-mentions the false positive rate is fairly high.

**Table 2:** **Overall performance of literature features on human proteins**

**Molecular function**				
**Features**	**F-max**	**Precision**	**Recall**	**macro-AUC**
Baseline (Original)	0.094	0.055	0.327	0.680
Baseline (Enhanced)	0.064	0.036	0.322	0.701
Co-mentions (Original)	0.386	0.302	**0.533**	0.769
Co-mentions (Enhanced)	0.377	0.336	0.447	0.764
BoW	0.394	**0.376**	0.414	0.768
Co-mentions + BoW	**0.408**	0.354	0.491	**0.790**
**Biological process**				
**Features**	**F-max**	**Precision**	**Recall**	**macro-AUC**
Baseline (Original)	0.134	0.091	0.249	0.610
Baseline (Enhanced)	0.155	0.103	0.311	0.611
Co-mentions (Original)	0.424	0.426	0.422	0.750
Co-mentions (Enhanced)	0.429	0.427	0.430	0.752
BoW	**0.461**	**0.467**	0.455	0.768
Co-mentions + BoW	0.459	0.426	**0.510**	**0.779**
**Cellular component**				
**Features**	**F-max**	**Precision**	**Recall**	**macro-AUC**
Baseline (Original)	0.086	0.050	0.305	0.640
Baseline (Enhanced)	0.073	0.041	0.317	0.642
Co-mentions (Original)	0.587	0.590	0.585	0.744
Co-mentions (Enhanced)	0.589	0.583	0.596	0.753
BoW	**0.608**	**0.594**	**0.624**	0.755
Co-mentions + BoW	0.607	0.592	0.622	**0.773**

Precision, Recall and F-max are micro-averaged across all proteins. Baseline corresponds to using only the co-mentions mined from the literature as a classifier. Macro-AUC is the average AUC per GO category. “Co-mentions + BoW” utilizes original co-mentions and BoW features within a single classifier.

### Performance on human proteins

We report performance of all four feature sets on human proteins in Table 2. Comparing the performance of the co-mention features, we find that the original co-mention features produce the better performance on Molecular Function (MF), while the enhanced co-mentions perform slightly better on both Biological Process (BP) and Cellular Component (CC). The most surprising result is that bag of words performed as well as it did, considering the complexity of the Gene Ontology with its many thousands of terms. Many text classification tasks utilize BoW and achieve very good performance while some have tried to recognize functional classes from text with BoW models with poorer results [19,20]. Their applicability to function prediction has only begun to be studied in this work and Wong *et al.* [6]. One explanation for their performance could be due to their higher utilization of the biomedical literature; co-mentions only capture information when both a protein and GO term are recognized together while BoW only relies on a protein to be recognized. In other words, the knowledge-based co-mentions are limited by the performance of automatic GO concept recognition, a challenging task in itself [16], while the BoW features have no such limitation. In support of that, we note that on average, there are 2,375 non-zero BoW features per protein, whereas there are an average of 135 sentence and 250 non-sentence non-zero co-mention features per protein. The results reported here are for human proteins; in Additional file 2 we provide results in yeast exhibiting the same trends observed in human.

Overall, best performance for all branches of the Gene Ontology is seen when using both co-mentions and the bag-of-words features. This suggests that all types of features provide complementary information. In view of this observation, we explored an alternative to using the features in combination to train a single classifier, which is to train separate classifiers and combine their scores. This approach gave similar results to those reported here (data not shown). It can be difficult to understand the impact of each type of feature solely by looking at the overall performance, since it is obtained by averaging across all proteins; we dive deeper in the following sections and provide examples that indicate that using co-mentions produces higher recall than precision.

Another observation to make is that performance for all three branches of GO as measured using the macro-AUC is very similar, indicating that the three sub-ontologies are equally difficult to predict from the literature. The differences in performance as measured by F-max, which is micro-averaged, are likely the result of the differences in the distribution of terms across the different levels in the three sub-ontologies. The similar performance across the sub-ontologies is in contrast to what is observed when using other types of data: MF accuracy is typically much higher than BP accuracy, especially when using sequence data [1,8], with the exception of network data such as protein-protein interactions that yields better performance in BP.

### Exploring differences between original and enhanced co-mentions

Examining Table 1, we see that the enhanced dictionary finds ∼35% (∼56 million) more unique co-mentions, makes about 32,000 fewer predictions (Table 3) and performs slightly better at the function prediction task (Table 2). To elucidate the differences that GO term recognition plays in the function prediction task, co-mention features and predictions were examined for individual proteins.

**Table 3:** **Description of the gold standard human annotations and predictions made by GOstruct from each type of feature**

	**Molecular**	**Biological**	**Cellular**
	**function**	**process**	**component**
**Feature type**	**# Predictions**	**# Predictions**	**# Predictions**
Gold standard	36,349	264,631	79,631
Original	102,486	268,068	76,513
Enhanced	64,919	276,734	81,094
BoW	40,499	268,114	77,753
Combined	62,039	386,267	78,475

All numbers are counts based on the predictions broken down by sub-ontology; these counts have the ‘true path rule’ applied.

Examining individual predictions it appears that many of the predictions made from enhanced co-mention features are more specific than both the original dictionary and the gold standard annotations; this is also supported by further evidence presented in the functional analysis in the *Functional class analysis* and *Analysis of individual Biological Process and Molecular Function classes* sections. For example, in GOstruct predictions using the original dictionary, DIS3 (Q9Y2L1) is (correctly) annotated with rRNA processing (GO:0006364). Using co-mentions from the enhanced dictionary, the protein is predicted to be involved with maturation of 5.8S rRNA (GO:0000460), a direct child of rRNA processing. There are 10 more unique sentence and 31 more unique non-sentence GO term co-mentions provided as features by the enhanced dictionary. Some of the co-mentions identified by the enhanced and not by the original dictionary refer to “mRNA cleavage”, “cell fate determination”, and “dsRNA fragmentation”. Even though none of these co-mentions directly correspond to the more specific function predicted by GOstruct, it could be that the machine learner is utilizing this extra information to make more specific predictions. Interestingly, the human DIS3 protein is not currently known to be involved with the more specific process, but the yeast DIS3 protein is. We did not attempt to normalize proteins to specific species because that is a separate problem in itself. It is probable that if we normalized protein mentions to specific species or implemented a cross-species evaluation utilizing homology the results of the enhanced dictionary would show improved performance.

We expected to see a bigger increase in performance because we are able to recognize more specific GO terms utilizing the enhanced dictionary. One possible reason that we don’t is due to increased ambiguity in the dictionary. In the enhanced dictionary, for example, a synonym of “implantation” is added to the term “GO:0007566 - embryo implantation”. While a majority of the time this synonym correctly refers to that GO term, there are cases such as “…tumor cell implantation” for which an incorrect co-mention will be added to the feature representation. These contextually incorrect features could limit the usefulness of those GO terms and result in noisier features. One way to address this may be to create a separate feature set of only co-mentions based on synonyms so the machine learner could differentiate or weight them differently; this could help improve performance using the enhanced dictionary co-mentions.

### Functional class analysis

**Figure 3:**
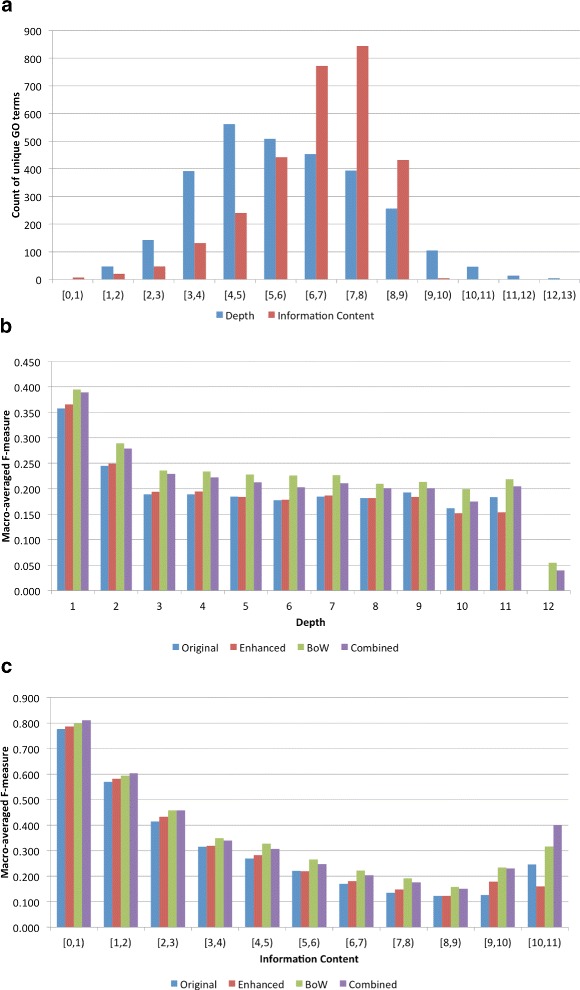
**Functional class analysis of all GO term annotations and predictions.** **a)** Distribution of the depth and information content of GO term annotations. As IC values are real numbers, they are binned, and each bar represents a range, e.g. ‘[1,2)’ includes all depth 1 terms and IC between 1 and 2 (not including 2). **b)** Macro-averaged F-measure performance broken down by GO term depth. **c)** Macro-averaged F-measure performance binned by GO term information content.

We now move to an analysis of functional classes to assess how well different parts of GO are predicted by different feature sets (Figure 3). We use two separate metrics, depth within the GO hierarchy and information content (IC) of the GO term derived from our gold standard annotations. Because the GO is a graph with multiple inheritance and depth can be a fuzzy concept [21], we define depth as the length of the shortest path from the root to the term in the GO hierarchy. We calculate an annotation-based information content(IC) for each GO term based on the gold standard annotations using the IC statistic described in Resnik *et al.* [22].

Figure 3(a) shows the distribution of counts of GO terms within the gold standard and predictions by both depth and information content, Figure 3(b) shows the macro-averaged performance (F-measure) for each feature set by depth, and Figure 3(c) shows the macro-averaged performance for each feature set binned by GO term information content. Examining 3(a) we find that terms appear to be normally distributed with mean depth of 4. Looking at information content, we find that over two-thirds of the terms have an information content score between 6 and 8, indicating that a majority of terms within the gold standard set are annotated very few times. Overall, for all sets of features, performance of concepts decreases as the depth and information content increases; it is intuitive that terms that are more broad, and less informative, would be easier to predict than terms that are specific and more informative.

Examining performance by depth (Figure 3(b)) we see a decrease in performance between depths 1-3, after which performance levels off. As a function of information content we obtain a more detailed picture, with a much larger decrease in performance with increased term specificity; all features are able to predict low information content, less interesting terms, such as “binding” (IC=0.20) or “biological regulation” (IC=0.66) with high confidence (F-measure > 0.8). Performance drops to its lowest for terms that have information content between 7 and 9 indicating there still remains much work to be done to accurately predict these specific and informative terms. Interestingly, there is an increase in performance for the most specific terms, especially using the BoW and combined representations; however, there are very few such terms as seen in (Figure 3(a)), representing very few proteins, so it’s not clear if this is a real trend. Finally, we observe that for both depth and IC analysis the knowledge-free BoW features usually outperform the knowledge-based co-mentions and that the enhanced co-mentions usually produce slightly better performance than the original co-mentions.

### Analysis of individual biological process and molecular function classes

To further explore the impact of the different features on predictions, we examined the best (Table 4) and worst (Table 5) Biological Process and Molecular Function categories.

**Table 4:** **Top biological process and molecular function classes predicted by each type of feature**

**Original co-mentions**							
**GO ID**	**Name**	**# Predictions**	**Precision**	**Recall**	**F-measure**	**Depth**	**IC**
GO:0009987	cellular process	6,164	0.812	0.875	0.842	1	0.66
GO:0044699	single-organism process	4,849	0.743	0.765	0.754	1	0.96
GO:0044763	single-organism cellular process	4,295	0.681	0.714	0.697	2	1.20
GO:0008152	metabolic process	3,893	0.644	0.726	0.682	1	1.22
GO:0065007	biological regulation	3,615	0.691	0.629	0.658	1	0.90
GO:0071704	organic substance metabolic process	3,489	0.611	0.677	0.643	2	1.42
GO:0050789	regulation of biological process	3,350	0.668	0.601	0.633	2	0.97
GO:0044238	primary metabolic process	3,337	0.593	0.655	0.623	2	1.56
GO:0044237	cellular metabolic process	3,268	0.590	0.644	0.616	2	1.49
GO:0050794	regulation of cellular process	3,156	0.648	0.583	0.614	3	1.11
GO:0050896	response to stimulus	2,968	0.606	0.590	0.597	1	1.62
GO:0043170	macromolecule metabolic process	2,640	0.548	0.618	0.581	3	1.77
**Enhanced co-mentions**							
**GO ID**	**Name**	**# Predictions**	**Precision**	**Recall**	**F-measure**	**Depth**	**IC**
GO:0009987	cellular process	6,223	0.816	0.887	0.850	1	0.66
GO:0007076	mitotic chromosome condensation	6	0.833	0.714	0.769	4	8.58
GO:0006323	DNA packaging	6	0.833	0.714	0.769	3	7.81
GO:0044699	single-organism process	4,957	0.744	0.783	0.763	1	0.96
GO:0044763	single-organism cellular process	4,423	0.682	0.736	0.708	2	1.20
GO:0008152	metabolic process	3,887	0.643	0.723	0.681	1	1.22
GO:0065007	biological regulation	3,701	0.683	0.636	0.659	1	0.90
GO:0050789	regulation of biological process	3,453	0.662	0.613	0.637	2	0.97
GO:0071704	organic substance metabolic process	3,491	0.605	0.670	0.636	2	1.42
GO:0043252	sodium-independent organic anion transport	11	0.636	0.583	0.608	7	8.50
GO:0000398	mRNA splicing, via spliceosome	140	0.492	0.697	0.577	10	5.88
GO:0006607	NLS-bearing protein import into nucleus	15	0.533	0.571	0.551	6	8.50
**Bag-of-words**							
**GO ID**	**Name**	**# Predictions**	**Precision**	**Recall**	**F-measure**	**Depth**	**IC**
GO:0009987	cellular process	6,005	0.820	0.869	0.844	1	0.66
GO:0044699	single-organism process	4,940	0.754	0.799	0.776	1	0.96
GO:0044763	single-organism cellular process	4,449	0.696	0.764	0.728	2	1.20
GO:0043252	sodium-independent organic anion transport	8	0.875	0.583	0.700	7	8.50
GO:0065007	biological regulation	3,865	0.698	0.686	0.692	1	0.90
GO:0008152	metabolic process	3,870	0.647	0.733	0.688	1	1.22
GO:0050789	regulation of biological process	3,597	0.680	0.663	0.671	2	0.97
GO:0006479	protein methylation	13	0.615	0.727	0.666	8	6.52
GO:0051568	histone H3-K4 methylation	13	0.615	0.727	0.666	11	7.94
GO:0007076	mitotic chromosome condensation	5	0.800	0.571	0.666	4	8.58
GO:0050794	regulation of cellular process	3,440	0.657	0.651	0.654	3	1.11
GO:0006497	protein lipidation	9	0.889	0.500	0.640	7	6.79
**Co-mentions + Bag-of-words**							
**GO ID**	**Name**	**# Predictions**	**Precision**	**Recall**	**F-measure**	**Depth**	**IC**
GO:0009987	cellular process	6,420	0.813	0.913	0.860	1	0.66
GO:0044699	single-organism process	5,338	0.736	0.834	0.782	1	0.96
GO:0044763	single-organism cellular process	4,862	0.674	0.800	0.731	2	1.20
GO:0065007	biological regulation	4,445	0.669	0.749	0.707	1	0.90
GO:0008152	metabolic process	4,252	0.638	0.785	0.704	1	1.22
GO:0050789	regulation of biological process	4,199	0.650	0.733	0.689	2	0.97
GO:0050794	regulation of cellular process	4,046	0.626	0.723	0.671	3	1.11
GO:0043252	sodium-independent organic anion transport	15	0.600	0.750	0.667	7	8.50
GO:0071704	organic substance metabolic process	3,883	0.602	0.743	0.665	2	1.42
GO:0043170	macromolecule metabolic process	3,007	0.540	0.694	0.607	3	1.77
GO:0051716	cellular response to stimulus	3,176	0.520	0.674	0.587	3	1.89
GO:0006386	termination of RNA polymerase III transcription	12	0.583	0.583	0.583	7	8.18

**Table 5:** **Most difficult biological process and molecular function classes**

**Original co-mentions**						
**GO ID**	**Name**	**# Predictions**	**Precision**	**Recall**	**F-measure**	**IC**
GO:0051179	localization	28	0.107	0.054	0.072	5.70
GO:0016247	channel regulator activity	115	0.043	0.208	0.071	6.53
GO:0009055	electron carrier activity	108	0.03	0.111	0.055	6.94
GO:0007067	mitosis	23	0.043	0.031	0.036	7.54
GO:0042056	chemoattractant activity	53	0.018	0.067	0.029	7.56
**Enhanced co-mentions**						
**GO ID**	**Name**	**# Predictions**	**Precision**	**Recall**	**F-measure**	**IC**
GO:0009055	electron carrier activity	102	0.090	0.138	0.109	6.94
GO:0051179	localization	42	0.071	0.055	0.061	5.70
GO:0019838	growth factor binding	44	0.021	0.035	0.027	5.99
GO:0070888	E-box binding	99	0.010	0.066	0.019	7.49
GO:0030545	receptor regulator activity	152	0.007	0.020	0.010	7.63
**Bag-of-words**						
**GO ID**	**Name**	**# Predictions**	**Precision**	**Recall**	**F-measure**	**IC**
GO:0051179	localization	18	0.277	0.090	0.137	5.70
GO:0009055	electron carrier activity	29	0.103	0.083	0.092	6.94
GO:0016042	lipid catabolic process	26	0.076	0.054	0.063	5.80
GO:0015992	proton transport	15	0.066	0.047	0.055	7.29
GO:0005516	calmodulin binding	14	0.071	0.033	0.045	7.25
**Co-mentions + Bag-of-words**						
**GO ID**	**Name**	**# Predictions**	**Precision**	**Recall**	**F-measure**	**IC**
GO:0051179	localization	61	0.100	0.109	0.104	5.70
GO:0009055	electron carrier activity	62	0.079	0.138	0.101	6.94
GO:0030545	receptor regulator activity	63	0.064	0.080	0.071	7.63
GO:0042056	chemoattractant activity	24	0.041	0.066	0.051	7.56
GO:0040007	growth	27	0.030	0.066	0.047	7.33

IC represents information content of term.

Examining the top concepts predicted, it is reinforced that the enhanced co-mentions are able to make more informative predictions, in addition to increasing recall without a loss in precision when compared to the original co-mentions. All 12 of the top terms predicted by the original co-mentions have an information content < 2 as opposed to only 7 terms from the enhanced co-mentions. We can compare the performance on specific functional classes. For example, “GO:0007076 - mitotic chromosome condensation” is the second highest predicted GO term by the enhanced co-mentions (F=0.769) while it is ranked 581 for the original co-mentions (F=0.526). Granted, there will always be specific cases where one performs better than the other; from these and previous analyses, we find that the enhanced co-mentions are able to predict more informative terms for more proteins than the original co-mention features (Figure 3 and Table 4). This shows that improving GO term recognition leads to an improvement in the specificity of function prediction.

Considering the top concepts predicted by the BoW features, we see a pattern similar to the enhanced co-mentions. Five out of the top twelve concepts predicted have an information content score greater than 6; these informative terms are different between the two feature sets. For the top functions predicted by all features the combined classifier of co-mentions and BoW produces more predictions, leading to higher recall and better F-measure. Even though some of the top terms predicted are informative and interesting we still strive for better performance on the most informative terms.

We also analyze the most difficult functional classes to predict, results can be seen in Table 5. Between all features we find some similar terms are difficult to predict; “localization” and “electron carrier activity” are in the worst five from all feature sets. It is interesting to note that the information content of these difficult to predict terms lies around the median range for all predicted terms. We might have expected that the most difficult terms to predict would be those most informative terms (IC around 10). We believe that these terms are difficult to predict because the ontological term names are made up of common words that will be seen many times in the biomedical literature, even when not related to protein function. This ambiguity likely results in a high number of features corresponding to these terms which results in poor predictive performance. There is still further work needed to address these shortcomings of literature mined features.

### Manual analysis of predictions

#### Manual analysis of individual predictions

We know that GO annotations are incomplete and therefore some predictions that are classified as false positives could be actually correct. The prediction may even be supported by an existing publication, however due to the slow process of curation they are not yet in a database. We manually examined false positive predictions that contain sentence level co-mentions of the protein and predicted function to identify a few examples of predictions that look correct but are counted as incorrect:

- Protein GCNT1 (Q02742) was predicted to be involved with carbohydrate metabolic process (GO:0006959). In PMID:23646466 [23] we find “Genes related to **carbohydrate metabolism** include PPP1R3C, B3GNT1, and **GCNT1**…”.
- Protein CERS2 (Q96G23) was predicted to play a role in ceramide biosynthetic process (GO:0046513). In PMID:22144673 [24] we see “…**CerS2**, which uses C22-CoA for **ceramide synthesis**…”.

These are just two examples taken from the co-mentions, but there are most likely more, which could mean that the true performance of the system is underestimated. Through these examples we show how the input features can be used not only for prediction, but also for validation. This is not possible when using features that are not mined from the biomedical literature and illustrate their importance.

#### Manual analysis of functional classes

In the previous section we explored individual co-mentions that could serve as validation for an incorrect GOstruct prediction. In addition to this one-off analysis, we can label subsets of co-mentions pertaining to particular functional concepts for validation on a medium-throughput scale. To identify functional classes for additional exploration, all GO concepts were examined for three criteria: 1) their involvement in numerous co-mentions with human proteins 2) numerous predictions made with an overall average performance and 3) confidence in the ability to extract the concept from text. The concepts chosen for further annotation were GO:0009966 – “regulation of signal transduction”, GO:0022857 – “transmembrane transporter”, and GO:0008144 - “drug binding”. For each of these classes all human co-mentions were manually examined.

We identified 204 co-mentions between a human protein and “GO:0008144 - drug binding” (IC=6.63). Out of 204 co-mentions, 112 appeared to correctly related the concept with the protein (precision of 0.554). 61 unique proteins were linked to the correct 112 co-mentions. Of these, only 4 contained annotations of “drug binding” in GOA, while the other 57 are not currently known to be involved with “drug binding”. When we examined the predictions made by GOstruct for these proteins, unfortunately, none of them were predicted as “drug binding”. After further examination of the co-mentions, most appear to be from structure papers and refer to drug binding pockets within specific residues or domains of the proteins. It is unlikely that the specific drug could be identified from the context of the sentence and many refer to a proposed binding site with no experimental data for support.

The concept “GO:0022857 - transmembrane transporter” (IC=4.17) co-occurred with a human protein 181 different times. 69 co-mentions appeared to correctly relate the concept with the labeled protein (precision of 0.381). A total of 32 proteins could be annotated with this concept; out of the 32 only 6 are not already annotated with “transmembrane transporter” in GOA. When we examine the predictions made from the enhanced features, only 1 out of the 6 proteins are predicted to be involved with “transmembrane transporter”.

There were a total of 134 human co-mentions containing “GO:0009966 – regulation of signal transduction” (IC=3.30). 73 out of 134 co-mentions appeared to correctly relate the concept with the protein (precision of 0.543). A total of 58 proteins could be annotated based upon these co-mentions. 21 proteins already contain annotations conceptually related to “regulation of signal transduction”, while the other 37 proteins do not contain annotations related to “regulation of signal transduction”; the later could represent true but uncurated functions. When we examine the predictions made by GOstruct using the enhanced co-mention features, 9 out of those 37 proteins were predicted to be involved with “regulation of signal transduction”.

When a random subset of 1,500 human co-mentions were labeled it was found that ∼30% (441 out of 1,500) correctly related the labeled protein and GO term. By annotating co-mentions of specific functional concepts we see that these categories have a higher proportion of correct co-mentions than the random sample from all co-mention; there will also be some categories where performance of co-mentions is quite low. This information can be used in multiple different ways. If we are more confident that certain categories related to function can be extracted from co-mentions, we can use this information to inform the classifier by encoding the information into the input features. Additionally, we show the importance and ability of co-mentions to not only be used as input features, but also for validation and enhancing the machine learning results. We show that many of the predictions made by our system could possibly be correct, but just not curated in the gold standard annotations.

#### Impact of evaluation metric on performance

In our initial experiments, we required predictions and gold standard annotations to match exactly (data not shown), but we found, through manual examination of predictions, that many false positives are very close (in terms of ontological distance) to the gold standard annotations. This type of evaluation measures the ability of a system to predict functions exactly, at the correct specificity in the hierarchy, but it doesn’t accurately represent the overall performance of the system. It is preferable to score predictions that are close to gold standard annotations higher than a far distant prediction. We are aware of more sophisticated methods to calculate precision and recall that take into account conceptual overlap for hierarchical classification scenarios [25,26]. For the results reported in Table 2, to take into account the hierarchy of the Gene Ontology, we expanded both the predictions and annotations via the ‘true path rule’ to the root. By doing this, we see a large increase in both precision and recall of all features; this increase in performance suggests that many of the predictions made are close to the actual annotations and performance is better than previously thought. A downside of our chosen comparison method is that many false positives could be introduced via an incorrect prediction that is of a very specific functional class. This could possibly explain why co-mentions from the enhanced dictionary display a decrease in performance; a single, very specific, incorrect prediction introduces many false positives.

## Conclusions

In this work we explored the use of protein-related features derived from the published biomedical literature to support protein function prediction. We evaluated two different types of literature features, ontology concept co-mentions and bag-of-words, and analyzed their impact on the function prediction task. Both types of features provided similar levels of performance. The advantage of the bag-of-words approach is its simplicity. The additional effort required to identify GO term mentions in text pays off by offering users the ability to validate predictions by viewing the specific literature context from which an association is derived, as demonstrated in our experiments.

In addition, we compared the value of concept co-mentions considering two different spans of co-occurrence: within a sentence (“sentence co-mention”) and across a sentence boundary (sentence-external, or “non-sentence co-mention”). Interestingly, we found that sentence and non-sentence co-mentions are equally useful, and that they are best used in conjunction as separate feature sets. Combining co-mentions and bag-of-words data provided only a marginal advantage, and in future work we will explore ways to obtain better performance from these features together. We also show that increasing the ability to recognize GO terms from biomedical text leads to more informative functional predictions. Additionally, the literature data we used provides performance that is on par with other sources of data such as network and sequence and has the advantage of being easy to verify on the basis of the text.

Our experiment in medium-throughput manual inspection of protein-GO term co-mentions suggests that this strategy can be used as a way of speeding up the process of curation of protein function. The literature contains millions of co-mentions, and a human-in-the-loop system based on the detected co-mentions prioritized by GOstruct can be a highly effective method to dramatically speed up the rate at which proteins are currently annotated.

### Future work

This work marks only the beginning of incorporating text mining for protein function prediction. There are always other more sophisticated or semantic features to explore, but based upon these results, there are some natural next steps.

The first would be to incorporate larger spans for a bag-of-words model due to the surprising performance of the non-sentence co-mentions. By including words from surrounding sentences, or an entire paragraph, more context would be en-coded and the model might result in better predictions.

Secondly, we found that an enhanced dictionary produced more individual co-mentions and fewer predictions, resulting in slightly increased performance. We explored several possible explanations as to why there is not a greater impact. It could be due to a large number of competing co-mentions that prevent good patterns from emerging or the possibility of introducing noise through ambiguous protein mentions. A filter or classifier that could identify a “good” co-mention would be providing much higher quality co-mentions as input, which would in turn likely lead to better predictions. Another way to potentially improve performance is to separate co-mentions found from synonyms from the original co-mentions, thereby allowing the classifier to provide them with different weights.

## Additional files

Additional file 1
**ConceptMapper parameters.** A description of the parameter values and impact for GO extraction through ConceptMapper.

Additional file 2
**Yeast results.** Function prediction performance numbers for prediction on yeast proteins.
