# Supplementary material for: Evaluating a variety of text-mined features for automatic protein function prediction with GOstruct
Source: J Biomed Semantics. 2015 Mar 18;6:9. doi: 10.1186/s13326-015-0006-4 (PMC4441003; doi:10.1186/s13326-015-0006-4)
Supplement: Additional file 1 — ConceptMapper parameters. A description of the parameter values and impact for GO extraction through ConceptMapper. [file 13326_2015_6_MOESM1_ESM.pdf]

Concept Mapper		
**Params**	**Description**	**Used for GO Extraction in this manuscript**
searchStrategy	CONTIGUOUS - returns longest match of contiguous tokens in the span, SKIP_ANY - returns longest match of not-necessarily contiguous tokens in the span, SKIP_ANY_ALLOW_OVERLAP - returns longest match of not-necessarily contiguous tokens in the span, this implies orderIndLookup	CONTIGUOUS
caseMatch	IGNORE - fold everything to lowercase more matching, INSENSITIVE - fold only tokens with initial caps to lowercase, SENSITIVE - performs no case folding, FOLD_DIGIT -fold only (and only) tokens with a digit	CASE_INSENSITIVE
stemmer	specifices which stemmer to use - PORTER, BIOLEMMATIZER, or NONE	PORTER
stopWords	a list of stopwords to remove - PUBMED or NONE	NONE
orderIndLookup	if set to TRUE token ordering within the sentence is ignored ("box top" would match "top box") - TRUE or FALSE	FALSE
findAllMatches	If TRUE all dictionary matches within the sentence are returned, otherwise only the longest is returned - TRUE or FALSE	TRUE
synonyms	specifies which synonyms will be included when making the dictionary - EXACT_ONLY or ALL	ALL
