# Supplementary material for: Evaluating a variety of text-mined features for automatic protein function prediction with GOstruct
Source: J Biomed Semantics. 2015 Mar 18;6:9. doi: 10.1186/s13326-015-0006-4 (PMC4441003; doi:10.1186/s13326-015-0006-4)
Supplement: Additional file 2 — Yeast results. Function prediction performance numbers for prediction on yeast proteins. [file 13326_2015_6_MOESM2_ESM.pdf]

Additional File 2 - Comparison of feature performance on yeast proteins

Here we present the raw data from predictions on yeast proteins. A majority of the conclusions reached on human proteins are also the same for yeast, the differences are pointed out within the main manuscript.

Ontology	Data source	F-max	Precision	Recall	micro-AUC	macro-AUC
Molecular Function	original (sent,non-sent)	0.594	0.547	0.661	0.992	0.894
	enhanced (sent,non-sent)	0.589	0.544	0.647	0.992	0.892
	bow	0.622	0.565	0.695	0.993	0.905
	combined (original,bow)	0.629	0.608	0.664	0.994	0.914
Biological Process	original (sent,non-sent)	0.587	0.526	0.664	0.982	0.880
	enhanced (sent,non-sent)	0.589	0.527	0.668	0.981	0.875
	bow	0.634	0.610	0.663	0.981	0.885
	combined (original,bow)	0.625	0.573	0.688	0.984	0.895
Cellular Component	original (sent,non-sent)	0.693	0.682	0.706	0.983	0.873
	enhanced (sent,non-sent)	0.695	0.699	0.692	0.984	0.874
	bow	0.719	0.706	0.733	0.984	0.875
	combined (original,bow)	0.712	0.689	0.737	0.986	0.892

Table 1 - Overall performance for all feature sets broken down by sub-ontology. Here we present overall performance and find that BoW and combined features are best performing and the enhanced co-mentions performs slightly better than the original co-mentions.

Ontology	Features	F-max	Precision	Recall	micro-AUC	macro-AUC
Molecular Function	Combined count	0.594	0.544	0.654	0.991	0.883
	sentence, non-sentence	0.594	0.547	0.661	0.992	0.894
	sentence	0.581	0.531	0.641	0.990	0.873
	non-sentence	0.576	0.521	0.643	0.991	0.879
Biological Process	Combined count	0.576	0.538	0.633	0.979	0.863
	sentence, non-sentence	0.587	0.526	0.664	0.982	0.880
	sentence	0.563	0.545	0.587	0.977	0.851
	non-sentence	0.570	0.568	0.571	0.978	0.867
Cellular Component	Combined count	0.687	0.703	0.672	0.979	0.851
	sentence, non-sentence	0.693	0.682	0.706	0.983	0.873
	sentence	0.683	0.687	0.679	0.978	0.841
	non-sentence	0.684	0.695	0.674	0.979	0.846

Table 2 – Experimenting with different ways to combine the sentence and non-sentence co-mentions from the original dictionary.

Ontology	Features	F-max	Precision	Recall	micro-AUC	macro-AUC
Molecular Function	Combined count	0.583	0.530	0.647	0.991	0.882
	sentence, non-sentence	0.589	0.544	0.647	0.992	0.892
	sentence	0.568	0.515	0.632	0.990	0.871
	non-sentence	0.567	0.511	0.636	0.991	0.878
Biological Process	Combined count	0.576	0.576	0.576	0.979	0.867
	sentence, non-sentence	0.589	0.527	0.668	0.981	0.875
	sentence	0.563	0.528	0.613	0.976	0.850
	non-sentence	0.570	0.516	0.648	0.978	0.865
Cellular Component	Combined count	0.689	0.704	0.674	0.979	0.846
	sentence, non-sentence	0.695	0.699	0.692	0.984	0.874
	sentence	0.686	0.696	0.675	0.979	0.843
	non-sentence	0.684	0.702	0.666	0.979	0.844

Table 3 – Experimenting with different ways to combine the sentence and non-sentence co-mentions from the enhanced dictionary.
